# Bovine proteins containing poly-glutamine repeats are often polymorphic and enriched for components of transcriptional regulatory complexes

**DOI:** 10.1186/1471-2164-11-654

**Published:** 2010-11-23

**Authors:** Vicki Whan, Matthew Hobbs, Sean McWilliam, David J Lynn, Ylva Strandberg Lutzow, Mehar Khatkar, William Barendse, Herman Raadsma, Ross L Tellam

**Affiliations:** 1 CSIRO Livestock Industries, Queensland Bioscience Precinct, 306 Carmody Rd, St Lucia, Queensland 4067, Australia; 2 Centre for Advanced Technologies in Animal Genetics and Reproduction (ReproGen), The University of Sydney, PMB3 Camden, NSW 2570, Australia; 3 Animal Bioscience Department, Teagasc, Dunsany, County Meath, Ireland

## Abstract

**Background:**

About forty human diseases are caused by repeat instability mutations. A distinct subset of these diseases is the result of extreme expansions of polymorphic trinucleotide repeats; typically CAG repeats encoding poly-glutamine (poly-Q) tracts in proteins. Polymorphic repeat length variation is also apparent in human poly-Q encoding genes from normal individuals. As these coding sequence repeats are subject to selection in mammals, it has been suggested that normal variations in some of these typically highly conserved genes are implicated in morphological differences between species and phenotypic variations within species. At present, poly-Q encoding genes in non-human mammalian species are poorly documented, as are their functions and propensities for polymorphic variation.

**Results:**

The current investigation identified 178 bovine poly-Q encoding genes (Q ≥ 5) and within this group, 26 genes with orthologs in both human and mouse that did not contain poly-Q repeats. The bovine poly-Q encoding genes typically had ubiquitous expression patterns although there was bias towards expression in epithelia, brain and testes. They were also characterised by unusually large sizes. Analysis of gene ontology terms revealed that the encoded proteins were strongly enriched for functions associated with transcriptional regulation and many contributed to physical interaction networks in the nucleus where they presumably act cooperatively in transcriptional regulatory complexes. In addition, the coding sequence CAG repeats in some bovine genes impacted mRNA splicing thereby generating unusual transcriptional diversity, which in at least one instance was tissue-specific. The poly-Q encoding genes were prioritised using multiple criteria for their likelihood of being polymorphic and then the highest ranking group was experimentally tested for polymorphic variation within a cattle diversity panel. Extensive and meiotically stable variation was identified.

**Conclusions:**

Transcriptional diversity can potentially be generated in poly-Q encoding genes by the impact of CAG repeat tracts on mRNA alternative splicing. This effect, combined with the physical interactions of the encoded proteins in large transcriptional regulatory complexes suggests that polymorphic variations of proteins in these complexes have strong potential to affect phenotype.

## Background

In humans there are more than 40 relatively rare progressive neurodegenerative, neurological and neuromuscular diseases linked with repeat instability mutations [1-4]. A distinct subset of this group, which includes for example Huntington disease and spinocerebellar ataxias, is caused by polymorphic variation in trinucleotide repeats, typically CAG repeats, often present in the coding sequences of specific genes [1-3,5-8]. In this group of 11 diseases the repeats usually encode long poly-glutamine (poly-Q) tracts, although other poly-amino acid tracts also occur but at lower frequencies. These human diseases are caused by extreme expansions of the repeats, which adversely impact protein structure, often causing intracellular protein aggregation and altered protein function [1,3,7,9,10]. However, trinucleotide repeat tracts which are impure, but maintain the ability to encode poly-Q tracts, have less severe impacts on function in model systems. This suggests that repeat expansions influence both protein and mRNA structure and function [1,4,8,11].

The length of the expanded repeat is typically inversely related to the age of disease onset and directly related to the severity of the disease [6,11]. Moreover, there is often a tendency for the expanded repeat to further increase in size in subsequent generations thereby resulting in greater disease severity and earlier onset, a phenomenon called genetic anticipation [1]. Trinucleotide coding sequence repeats in many genes are often polymorphic in the normal population but these repeats are typically shorter in length compared with disease-causing alleles. In some instances the distinction between normal and disease states can be minor in terms of repeat length [1,11].

In a restricted examination of 7,039 human, rat and mouse orthologs 75, 53 and 58 genes, respectively, containing poly-Q encoding CAG repeats (CAG poly-Q genes) were identified [12]. Another analysis of the human genome reported a total of 66 CAG poly-Q genes [11]. These regions were defined as containing five or more CAG repeats encoding poly-Q. Although poly-Q containing proteins are generally more highly conserved in mammals, paradoxically their repeat regions are typically poorly conserved both in sequence and length, and they have been implicated in the rapid functional diversification of these proteins and the continuous morphological evolution of mammals [13-15]. It has also been suggested that normal polymorphic variation in these genes contributes to morphological variation within a species, as many of the affected genes are involved in regulating aspects of development and often encode transcription factors and transcription co-regulators [11,14]. Indeed, polymorphic trinucleotide repeats in two genes, *RUNX2 *(runt-related transcription factor 2) and *ALX4 *(aristaless-like homeobox 4), have been linked with morphological variation in canine skulls and limbs [15,16]. It is possible therefore that those genes containing polymorphic coding sequence trinucleotide repeats may contribute to variation in a broad range of phenotypes within a species.

What is not clear is whether the propensity for normal polymorphic variation in the CAG repeats of some of these genes also occurs in non-primates. There is significant positive correlation between the length of a pure CAG repeat and its propensity for polymorphic variation in human genes [11]. Interruptions of the CAG repeat, even with the alternate codon for glutamine, CAA, decrease the likelihood of polymorphic variation, as do reading frame shifts maintaining the CAG repeat structure but encoding different amino acids [11]. Moreover, the potential for a gene to cause known human diseases was also associated with greater CAG repeat polymorphic diversity [17]. Thus, it is probable that long and pure CAG tracts encoding poly-Q are predictors of polymorphic variation that is likely to impact on phenotype.

Identification of poly-Q encoding genes in additional mammalian species may identify species-specific differences in both the repertoire of these genes as well as the diversity of polymorphic variants in a subgroup of these genes. The former genes may contribute to an understanding of morphological and functional differences between mammalian species while the latter may contribute to phenotypic variation within a species. The current investigation has identified all bovine poly-Q encoding genes and within this group a number of genes whose orthologs in human and mouse do not contain these repeats. The bovine poly-Q encoding genes were prioritised using multiple criteria for their likelihood of being polymorphic and then the highest ranking group was experimentally tested for polymorphic variation within a cattle diversity panel. Extensive polymorphic variation was identified. The poly-Q encoding genes were strongly enriched for molecular functions relating to transcriptional regulation and their encoded proteins are involved in large transcriptional regulatory complexes in the cell nucleus.

## Results

### Identification of poly-Q encoding bovine genes

Bovine genes encoding tracts of five or more glutamines (poly-Q tracts) were initially identified using the NCBI RefSeq collection [18] and GLEAN5 gene models in conjunction with the BTAU 3.1 bovine genome sequence assembly [19,20]. They were subsequently manually confirmed using the current Btau_4.0 assembly. A tract of five or more Q in a protein sequence has been identified as statistically different from the average protein with average composition [21]. A total of 178 poly-Q encoding genes were identified of which 36 contained multiple tracts and 123 contained pure CAG encoding poly-Q repeats (Additional file 1).

**Figure 1:**
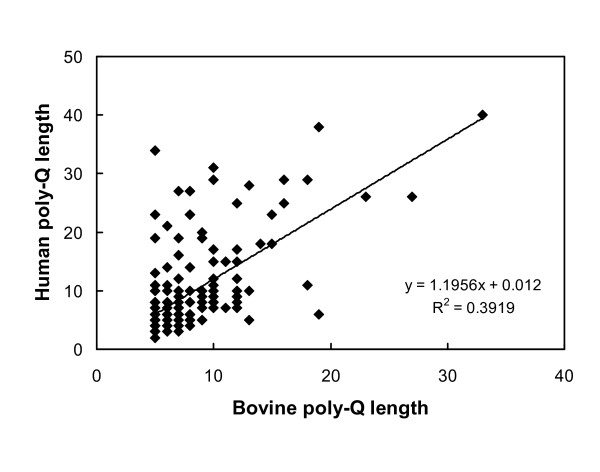
**Characteristics of bovine poly-Q proteins**. Comparison of the sizes of Q-tracts in bovine and human poly-Q orthologs.

The presence of a polymorphic repeat tract occasionally confused automated gene model annotation pipelines which utilised cDNA and genomic sequence information representing different alleles in the repeat region. For example, most gene models for bovine *FXC1 *(fractured callus 1) are incorrect as they attribute an additional intron to the repeat region, which clearly showed allelic differences between genomic DNA and EST sequences [22]. This annotation error is prevalent in other unrelated gene models where polymorphic repeats are prevalent (e.g. [23]). The mean poly-Q length encoded by the 178 genes was 7.26 ± 3.55 (1 sd). The longest tract, containing 33 Q residues, was present in FOXP2 (forkhead box P2) while MLL2 (myeloid/lymphoid or mixed lineage leukemia 2) contained the greatest number of repeat tracts, 19. A similar analysis revealed that the human genome contained 201 poly-Q encoding genes of which 109 are orthologs of bovine poly-Q encoding genes (result not shown). Figure 1 shows that most bovine poly-Q proteins have poly-Q tract sizes approximately similar to their human orthologs. Twenty six bovine poly-Q encoding genes were identified with Q-tracts that were not evident in their human and murine orthologs when examined using HomoloGene [18] (bolded in Additional file 1). The mean size of the bovine poly-Q proteins (1,034 ± 913 amino acids (1 s.d.; n = 155)) deduced from the longest Ensembl gene model information [22] was significantly larger than for all bovine proteins (mean = 365 ± 276 amino acids; 1 s.d.; n = 14,985; p < 0.001). Poly-Q tracts were not enriched in any one third of the protein sequence lengths (p > 0.05). Therefore, they were not biased toward the ends of the polypeptides where they may be expected to be better tolerated in terms of their impacts on polypeptide functions.

### Polymorphic variation of bovine poly-Q encoding genes

Several criteria have been associated with the propensity for polymorphic variation in poly-Q encoding genes [11]. Bovine poly-Q encoding genes were prioritised in a hierarchical manner according to the following criteria: (i) length and purity of CAG repeat; (ii) lack of conservation of CAG repeat length in mammalian orthologs; (iii) evidence for polymorphic variation in other mammalian species; (iii) evidence that polymorphic variations in the human or mouse orthologs caused diseases, and; (iv) disparities between bovine cDNA sequences and the BTAU4.0 reference genome sequence in regions of bovine genes encoding poly-Q tracts. The top ranked 32 genes were initially screened by PCR for polymorphic variation in amplicon size using a small diversity panel consisting of eight cattle. Five of these genes contained two poly-Q tracts and in each case both were independently assayed. In addition, three genes that were not highly ranked were also tested (i.e. *ODAM *(odontogenic ameloblast-associated protein), *AR *(androgen receptor) and *NFYA *(nuclear transcription factor Y, alpha)). The latter analysis was used as a control for the prioritization process. *ODAM *is situated within a cluster of casein genes and hence it is also of biological interest due to potential strong selection at this locus. Polymorphic poly-Q variants of human AR have been linked with a number of reproductive traits [24], while *NFYA *was particularly interesting as its poly-Q encoding tract traverses a splice site (see below).

**Figure 2:**
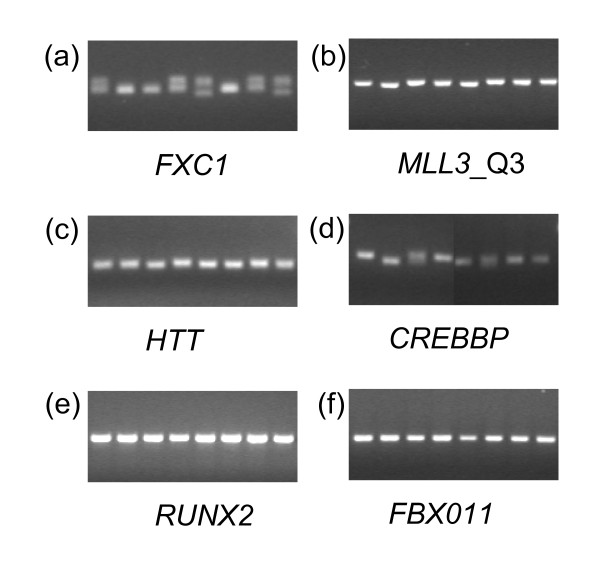
**Representative PCR amplicons for bovine poly-Q encoding genes**. Amplicons for a number of bovine poly-Q encoding genes were produced by PCR using genomic DNA derived from a small cattle diversity panel consisting of 8 individuals. Panels (a)-(d) highlight polymorphic genes, while panels (e) and (f) show examples of genes that were monomorphic. Each lane represents one unrelated individual. The lane order representing the 8 animals is the same for each gene. Amplicons were visualised on 3% agarose gels stained with ethidium bromide. More than one poly-Q encoding tract within a gene was signified by extension to the gene symbol (e.g. *MLL3*-Q3).

Of the total of 35 genes tested, 19 showed suggestive evidence for polymorphic variation in the initial 8 animal screen. Figure 2 shows representative profiles for a number of these genes as well as two (*RUNX2 *and *FBX011*) that showed no amplicon size variation in the small cattle diversity panel. *FXC1 *was the most polymorphic gene in the initial analysis.

**Figure 3:**
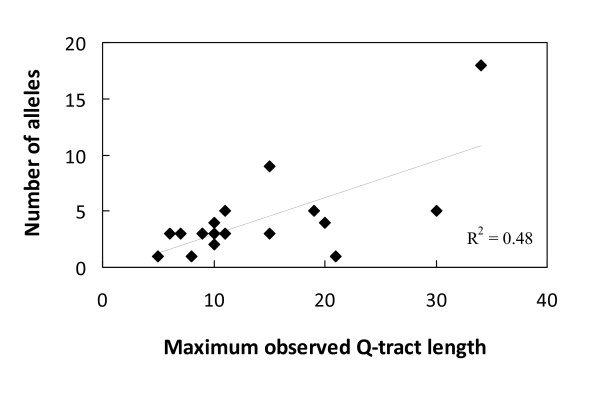
**Relationship between allelic diversity and the number of CAG-encoding glutamines within a repeat**. The number of alleles identified for each polymorphic bovine gene in the large cattle diversity panel was plotted against the number of poly-Q encoding CAG repeats present within the largest allele of each gene.

A larger cattle diversity panel consisting of 150 animals (Additional file 2) was then used for automated genotyping of the polymorphic regions in the 19 genes identified in the initial screen. The analysis also included poly-Q encoding regions within *ODAM*, *NFYA *and *AR*, all of which were found to be monomorphic in this larger cattle diversity panel, as they were in the initial small animal screen. The results of this analysis are presented in Table 1. A total of 16 of the 19 genes identified in the preliminary screen were confirmed as polymorphic in the large diversity panel, with nearly all (15/16), having more than two alleles. Representatives of each gene were sequenced to confirm the allelic variation. As anticipated from the preliminary screen, *FXC1 *was the most polymorphic gene with 18 alleles. Also of note were the nine *HTT *(Huntington) alleles. The latter gene is the prototypical CAG poly-Q gene in humans. Extreme repeat expansions in this gene are associated with the autosomal dominant neurological disorder, Huntington disease [1,6]. CAG repeat expansions or mutations in 4 of 16 human orthologs of the polymorphic bovine poly-Q genes are associated with diseases [1,3,8,11]. The mean size of the proteins encoded by the 16 bovine polymorphic genes was not different from all bovine poly-Q proteins (p > 0.05). Figure 3 shows that for these 16 genes there is a linear relationship between the largest number of repeats in an allele of a gene and the number of alleles. An independent analysis of these 16 genes in 82 Holstein dairy cattle, representing 28 trios, each consisting of both parents and one offspring, revealed stable inheritance of all 16 polymorphic genes (result not shown).

**Table 1:** Polymorphic variation in bovine poly-Q genes

Gene Symbol	**Gene Coodinates**^**1**^	**Q-tract**^**2**^	**Allele No**.	**Prob. Bt vs Bi**^**4**^	**Prob. beef vs dairy**^**5**^
*ABCF1*	BTA23: c28,330,331-28,317,176	G1A1G9A1	3	NS	NS
*C10ORF26*	BTA26: 23,731,487-23,787,266	G8	1	-	-
***CACNA1A***^6^	BTA7: 10,407,726-10,660,224	G6	3	< 0.05	< 0.01
*CREBBP*	BTA25: c3,741,183-3,622,474	G5A1G3A1G5	4	< 0.001	NS
*EXDL2*	BTA10: 83,270,819-83,301,240	G9A1	3	< 0.0001	NS
*FAM155A*	BTA12: c80,851,585-81,459,510	G10	6	< 0.0001	NS
*FAM48A*	BTA12:24,343,037-24,382,185	G1A1G6	3	< 0.0001	NS
*FXC1*	BTA15: c45,697,647-45,662,984	G10A1	18	< 0.0001	< 0.001
***HTT***^6^	BTA6: 120,080,853-120,204,565	G15	9	< 0.0001	<0.01
*LRCH4*	BTA25: 38,082,422-38,093,009	G7	3	< 0.0001	NS
***MED12***^6^	BTAX: c49,328,169-49,305,901	G6A1G2A1G1A	5	< 0.01	NS
		1G13A1G1N4G5			
*MED15*	BTA17: c75,807,030-75,798,191	G6NG1NG3N3G	3	< 0.001	NS
		10NG1NG2N3G			
		3NG1N3G5NG4			
*MEF2A*	BTA21: c5,782,758-5,605,728	G5	1	-	-
*MLL3*	BTA4: c119,034,986-118,841,623	G5A1	4	< 0.0001	NS
*NCOR1*	BTA19: 34,217,888-34,332,169	G10	2	< 0.0001	NS
*RUNX2*	BTA23: 19,237,338-19,466,747	G3A2G3A1G5A	1	-	-
		1G3A1G2			
*ST6GALNAC5*	BTA3: c72,520,937-72,308,057	G7	3	< 0.0001	NS
***TBP***^6^	BTAUN: WGA3026_4: 46,413-59,300	G5A1G13	4	< 0.0001	NS
*THAP11*	BTA18: 34,372,276-34,373,950	G5A1G4A1G1A	5	< 0.05	NS
		1G5			
***AR***^3^,^6^	BTAX: c51,674,166-51,881,948	G3A1G4;	1	-	-
		G3A1G4	1	-	-
*NFYA*^3^	BTA23: 15,621,415-15,645,446	G3;	1	-	-
		G2	1	-	-
*ODAM*^3^	BTA6: 88,472,908-88,481,760	A1G4	1	-	-

^1^BTAU4.0 bovine genome assembly (UCSC genome browser). BTA, chromosome number; UN, chromosome unknown; c, complementary strand.

^2^Length and purity of repeat triplet codon. G, A and N followed by a number represent CAG, CAA and other triplet codon tracts, respectively, that were present in the BTAU 4.0 bovine genome assembly. Multiple closely linked Q-tracts are shown as a single entity connected by Ns. Multiple discontinuous Q-tracts within a gene that were assayed are separated by a semi-colon. The Q-tract for NFYA traversed a splice site and is represented by two genomic regions each of which was genotyped.

^3^These genes were not ranked highly for potential polymorphic variation using the hierarchical prioritisation criteria.

^4^Probability of difference in allele frequencies between *Bos taurus taurus *and *Bos taurus indicus *animals (Fisher exact test [25]). NS, not significant.

^5^Probability of difference in allele frequencies between *Bos taurus taurus *beef and dairy breeds (Fisher exact test [25]). NS, not significant.

^6^Bolded gene symbols have been linked with human diseases caused by poly-Q tract expansions or mutations [53].

The allele frequencies in the large cattle diversity panel showed significant differences between *Bos taurus taurus *and *Bos taurus indicus *animals for 15 of 16 of the polymorphic poly-Q genes (p < 0.05; Fisher exact test, [25]) (Table 1 and Additional file 3). For some genes there was a clearly predominant allele (e.g. *MED12 *(mediator complex 12) and *THAP11 *(THAP domain containing 11)) while others showed broad distribution profiles (e.g. *FXC1 *and *HTT*) (Additional file 3). Allele frequencies of four genes, *CACNA1A *(calcium channel, voltage dependent P/Q type, alpha 1A subunit), *FXC1, HTT *and *TBP *(TATA box binding protein), were also different (p < 0.05; Fisher exact test) in *Bos taurus taurus *cattle breeds specialised for beef or dairy uses. Larger cattle populations will need to be assessed in subsequent studies to confirm these results and to also re-examine some of the highly ranked genes that were apparently monomorphic as the process used for gene selection was likely to exclude low frequency allelic variation.

### Enrichment of poly-Q encoding genes for functional terms

To test the hypothesis that particular functional classes of genes are enriched in the full repertoire of bovine poly-Q encoding genes we undertook gene function enrichment analyses. The Database for Annotation, Visualization and Integrated Discovery (DAVID) functional analysis tool was used to identify over-represented (p < 0.05; Benjamini correction for multiple testing) gene ontology (GO) terms, pathways and keywords [26,27]. DAVID was then used to group the significantly enriched terms into *Functional Annotation Clusters *as many functional terms were associated with overlapping gene contents. This process provided a higher level perspective of the enriched functions associated with these genes. There were 21 functional annotation clusters with enrichment scores greater than 1.3, which is considered a significance threshold. Additional file 4 contains the complete analysis. Cluster 1 was the most significant (enrichment score = 21.36) and it contained the most terms. This cluster was over-represented with many terms relating to control of gene transcription, as well as terms associated with regulation of metabolism and biological processes. Strikingly, Clusters 1, 5-7, 9-17, 19 and 20 all contained terms linked with transcriptional regulation and consistent with this there was corresponding enrichment for nuclear organelle structure (Clusters 2 and 4). As might be expected, there was enrichment for themes relating to trinucleotide repeats (Cluster 21). The analysis also highlighted metabolic processes (Clusters 1 and 3). Only one KEGG pathway was over-represented i.e. *The Notch Signalling Pathway *(P = 2.3E-3, Benjamini correction for multiple testing). Although not reaching significance after correction for multiple testing, *Neurodegenerative Diseases *and *Huntington's Disease *KEGG pathways were close to the significance threshold. In humans both of these pathways are closely associated with coding sequence CAG repeat expansions that generate diseases.

Human orthologs of the bovine poly-Q encoding genes were enriched for gene expression (UP_Tissue database) in *Epithelium *(p = 7.6E-13, after Benjamini correction for multiple testing), *Brain *(p = 7.1E-4, corrected for multiple testing) and *Testis *(p = 3.8E-2, corrected for multiple testing) (DAVID database). Manual examination of the expression of these genes using human and murine gene expression databases (GEO database; GDS182 (mouse) and GDS1402 (human); [18]) generally revealed ubiquitous expression patterns but with bias towards greater expression in epithelia. The 26 poly-Q genes unique to bovine (i.e. containing poly-Q tracts that were not present in mouse or human orthologs) were not significantly different from all of the bovine poly-Q encoding genes with respect to functional terms as measured by DAVID or by examination of gene expression patterns. Likewise, the 16 polymorphic genes were also not enriched for functional terms relative to all poly-Q encoding genes.

**Figure 4:**
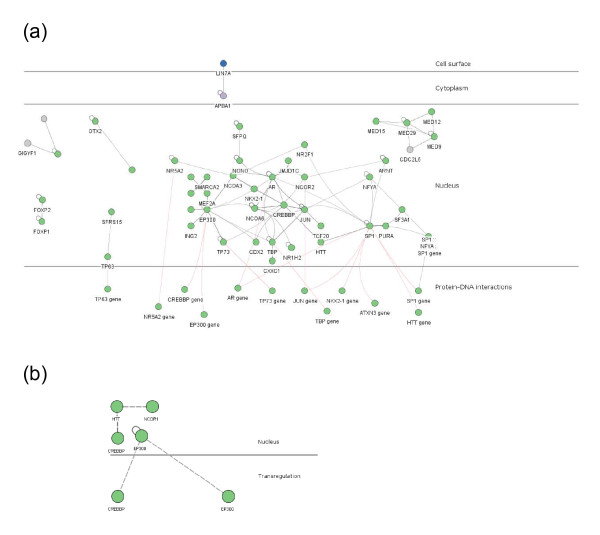
**Interactions of poly-Q proteins**. The Innate DB database was used to identify networks of poly-Q proteins that have been documented to physically interact with each other and relationships between the same group of proteins and transcriptional regulation of poly-Q encoding genes [28,29]. (a) Interaction network for bovine poly-Q proteins. Black lines signify documented physical interactions while red lines refer to transcriptional regulation. Singletons representing either self-interactions or cis-acting transcriptional regulation are not shown. The cell is partitioned into cell surface, cytoplasm and nucleus. Protein-gene linkages (red) are shown at the bottom of the diagram. (b) Interaction network for the identified polymorphic bovine poly-Q encoding genes.

The gene function analysis indicated strong enrichment for transcription factors and transcriptional co-regulators in the bovine poly-Q genes. Many transcription factors and their co-regulators physically interact to form functional units controlling gene expression. We therefore tested the extent of interactions for the proteins encoded by human orthologs of the bovine poly-Q genes by using the InnateDB database [28,29]. Figure 4(a) shows the network of human orthologs to the bovine poly-Q proteins that have been experimentally documented to be involved in physical interactions with other proteins from the same group. The diagram emphasises the nuclear location of many poly-Q proteins and that they have potentially extensive physical interactions within the nucleus. Some poly-Q proteins, such as CREBBP (cAMP response element binding (CREB) protein), EP300 (E1A binding protein p300) and SP1 (SP1 transcription factor) had more extensive heterologous interactions. In addition, the transcription of several genes in this group was also controlled, at least in part, by proteins present in the same group (red connections). Figure 4(b) shows a smaller interaction network involving only those proteins whose bovine orthologs were shown to be polymorphic. CREBBP and NCOR1 (nuclear receptor co-repressor 1) are both directly involved in physical associations with HTT [30,31].

### The impact of CAG repeat regions on mRNA splicing

**Figure 5:**
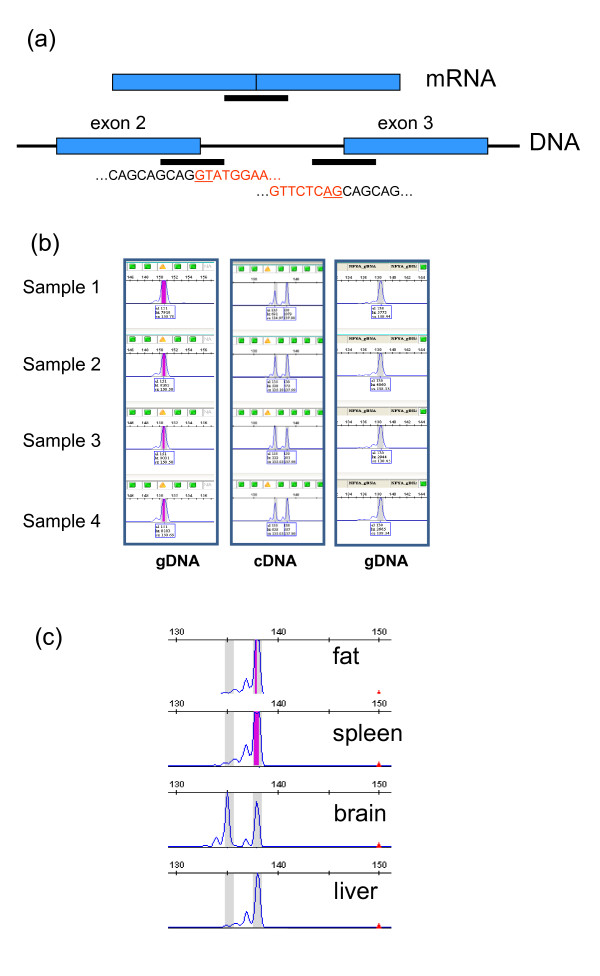
**Expression of *NYFA *mRNA**. (a) Diagrammatic representation of the *NFYA *genomic structure in relation to the poly-Q encoding region traversing the *NFYA *splice site across exons 2 and 3. The PCR amplicons corresponding to the exon 2-intron 2 and intron 2-exon 3 boundary regions when using genomic DNA (gDNA), and the amplicon produced when using cDNA as template are shown as thick black lines. The sequence at each splice boundary is shown with exonic sequence in black and intronic sequence in red. The absolutely conserved intronic sequence elements at the splice sites are underlined. (b) PCR amplicon profiles for four individuals. The left hand and right hand panels show the exon 2 - intron 2 and intron 3 - exon 3 amplicons, respectively, with genomic DNA as template. The central panel shows the amplicons produced using mammary tissue cDNA and primers traversing the splice site junction. Two amplicons encoding 4 or 5 Q were identified. (c) Tissue specific *NYFA *mRNA splicing across the poly-Q encoding region. Representative results are shown for one of four individuals for four different tissues.

During analysis of the bovine genome sequence we identified discordant ESTs associated with poly-Q encoding tracts of bovine genes, which subsequently led to the identification of a number of polymorphic variants. The same analysis also highlighted discordances potentially arising from alternative mRNA splicing events. NFYA is a ubiquitously expressed highly conserved transcription factor subunit that, in conjunction with NFYB and NFYC, binds to CCAAT motifs in the promoters of many genes that are expressed in a tissue specific manner [32]. Reverse transcriptase-PCR (RT-PCR) results demonstrated that NFYA was expressed in a broad spectrum of bovine tissues (result not shown). This result was confirmed by manual inspection of murine and human gene expression databases (GEO accessions GDS182 (mouse) and GDS1402 (human) [18]). Close inspection of the cDNA amplicons, which traversed the poly-Q encoding region, suggested that they were not all equivalent in size. Figure 5(a) shows a diagrammatic representation of the organisation of exons 2 and 3 of the *NFYA *gene and includes the sequences flanking the exon 2 - intron 2 and intron 2 - exon 3 boundaries. The poly-Q encoding region traversed the mRNA splice site i.e. three CAG codons were present in exon 2 and two in exon 3. Neither of these flanking regions was polymorphic in four individuals when genomic DNA was assayed by PCR (Figure 5(b)). Nor was this region polymorphic in the large cattle diversity panel. However, when cDNA from mammary tissue was used as template, two amplicons differing by three nucleotides were evident. When sequenced, these amplicons contained either four or five CAG codons in the poly-Q encoding region. Moreover, there were clear tissue-specific *NFYA *expression differences for these two variants (Figure 5(c)). Only the longer variant was expressed in fat, spleen and liver while both variants were expressed in mammary and brain tissues. Thus, different splice donor or acceptor sites in the CAG repeat region of the gene were used to generate tissue specific mRNA sequence variation. Since the N-terminal region of NFYA, and in particular its poly-Q segment, possesses significant transcriptional activation activity, then the splice site variation could be generating tissue specific differences in the activity of this transcription factor [33,34].

Examination of the gene models for *ABCF1 *(ATP-binding cassette, sub-family F, member 1) [22], one of the polymorphic poly-Q encoding genes, as well as *MLL2 *and *FOXP2*, revealed that their repeat regions also traversed splice sites. Moreover, several of the poly-Q encoding genes had repeats that were juxtaposed at or very close (< 5 bp) to splice sites (*ATXN3 *(ataxin 3), *FOXP1 *(forkhead box P1), *MED12 *(mediator complex subunit 12), *TNRC15 *(trinucleotide repeat containing 15), *TNRC4 *(trinucleotide repeat containing 4), *CXXC1 *(CXXC finger 1), *CDC2L6 *(cell division cycle 2 -like 6)*, ANKRD56 *(ankyrin repeat domain 56), *AMOT *(angiomoton), while some repeats were contained within small alternatively spliced exons (e.g. *CACNA1A, MED15 *(mediator complex subunit 15), *NCOR1; AMOT*). Thus, there is potential for poly-Q tracts and their allelic variants to influence alternative splicing through a number of possible mechanisms.

Additional file 5 shows UCSC bovine genome browser [22] representations of the 3' and 5' ends of two adjacent exons in *MED12*. The exons are separated by an ~ 820 bp intron. Both exons encode large poly-Q tracts. Also included is protein sequence information for cow, dog, human and mouse as well as informative bovine ESTs. It is evident from the ESTs that there is considerable transcriptional diversity being generated from these two exons. This diversity could comprise the effects of polymorphic variation as well as alternative splicing because the ESTs are derived from a number of individuals. However, within one library from a single individual there are a number of very different ESTs (DN274709, DN540107, DN518332, DN521072, DN538689). The splice donor and acceptor sites in all of these cases are situated within CAG repeat tracts suggesting a direct involvement of these tracts in generating transcriptional diversity through their influence on alternative splicing. Alternatively, there is a possibility that the transcriptional diversity reflects heterozygosity for polymorphic alleles within this individual. However, this explanation cannot account for the full scope of the transcriptional diversity.

## Discussion

The current study identified 178 bovine poly-Q encoding genes and demonstrated that a substantial number of the ranked genes were polymorphic in their repeat containing regions. Assessment of larger populations of cattle will be required to define the full repertoire of polymorphic poly-Q encoding genes as lower frequency alleles were probably excluded in the current analysis. The lack of conservation of the repeat regions associated with polymorphic variation in poly-Q encoding genes indicate that these are rapidly evolving gene regions [13]. Indeed, it has been estimated that the insertion-deletion mutation rate of microsatellites, such as CAG repeats in mammals, is approximately 100,000 fold greater than single nucleotide substitutions [14]. As alterations in the number of trinucleotide repeats do not cause frameshifts in coding sequences, variation in these regions both between and within species is probably much better tolerated than other types of indels. Longer CAG repeats with little interruption by the alternative glutamine encoding codon CAA, are more likely to be associated with higher allele diversity (Figure 3) [11,35-39]. Polymerase slippage, unequal crossing-over during replication, and repair associated mechanisms, are the most likely explanations for this increased genetic diversity [4,40,41]. The overall balance between the mutational mechanisms that promote repeat expansions and the accumulation of point mutations that decrease repeat purity and therefore decrease the probability of repeat expansion is likely to dictate the nature of the poly-Q tract within a gene and its propensity for allelic variation.

Although there are a number of human diseases that are caused by instability in the repeat regions of poly-Q encoding genes, particularly expansion in subsequent generations (anticipation) and during aging, there was no evidence for meiotic instability in the 16 polymorphic poly-Q encoding bovine genes that were tested. A similar result was also demonstrated for porcine *HTT *[42]. One likely explanation is that the identified bovine polymorphic gene variants represented normal alleles of relatively short lengths and not the extreme expansions characteristic of some poly-Q encoding genes that cause human diseases [1,6,11].

It has been argued that proteins containing repeats simply reflect the propensity for variation and that these regions are not subject to selection and have no functional impact [43,44]. These conclusions are at odds with substantial evidence demonstrating that amino acid repeat size in a wide spectrum of human genes impacts on molecular function and that these regions are indeed subject to strong selection [4,9,10,13,24,30,45-51]. In particular, Q-tracts have been shown to be autonomous length-dependent activators of DNA binding activities in transcription factors [46,47,49]. It is noted that there was strong enrichment for transcription factors in the bovine poly-Q encoding genes and thus it is likely that intra-species and inter-species poly-Q tract expansions (or contractions) directly impact the transcriptional activities of these proteins. This is an appealing concept as it links propensity for increased genetic diversity with subtle functional differences in a class of proteins that can amplify these genetic influences because they impact the transcriptional regulation of a much broader group of genes. Moreover, based on evidence linking normal polymorphic variations in two poly-Q encoding genes, *RUNX2 *and *ALX4*, with morphological variation in dogs, it has been proposed that poly-Q encoded genes contribute raw genetic material for rapid evolutionary change in a species [10,14,15,52].

Those genes containing conserved repeats, which often tend toward shorter and less pure repeat sequences, may reflect strong functional constraints on the encoded proteins in these regions and therefore selective pressure against variation despite increased mutational opportunity. Consistent with this concept, these genes are rarely polymorphic in their repeat regions within a species population [11]. This information highlights local regions which may have important functions. The absence of polymorphic variation in the poly-Q tract of the bovine androgen receptor (AR), a co-activator for androgen dependent gene transcription, is interesting in view of the links between poly-Q variation in the human protein and androgen-dependent diseases, many of which influence reproductive success [53,54]. As the *AR *gene is carried on the X chromosome some of these diseases result in somatic mosaicism [35]. The long history of cattle domestication involving intensive sire-based selection, primarily focussed on growth and reproductive traits, may have selected against variation in the *AR *gene in cattle.

Comparison of allele frequencies in *Bos taurus indicus *and *Bos taurus taurus *breeds showed significant differences for 15 of 16 polymorphic poly-Q encoding genes. Since the repeat regions in these genes are subject to relatively high rates of mutation, then these data could suggest differences in the microsatellite mutational rates within the two breeds. This possibility seems unlikely as there were no systematic effects on the repeat sizes or the number of alleles for each gene. An alternative explanation is that the allele frequency differences reflect variation that has independently arisen since divergence of these two cattle breeds from an ancestral population. The frequencies of alleles for four genes, *CACNA1A, FXC1, HTT *and *TBP *were significantly different for *Bos taurus taurus *cattle specialised for milk or meat production. All of these genes have ubiquitous expression patterns (GEO database accessions; GDS182 (mouse) and GDS1402 (human)). TBP (TATA box binding protein) and HTT are transcriptional co-regulators located in the nucleus, CACNA1A is a subunit of a plasma membrane voltage dependent calcium channel and, FXC1 is a component of a hetero-oligomeric translocase complex present in the mitochondrial inner membrane where it plays a role in the import of proteins into the inner mitochondrial membrane. Thus, structural variations that impact on the functions of these proteins have potential to modify broad transcriptional activities and metabolism. These influences may be subject to different selective pressures in beef and dairy cattle populations. Notably, extreme polymorphic repeats in three of four of these genes (*CACNA1A*, *HTT *and *TBP*) are associated with a spectrum of human diseases [53].

*FXC1 *was the most polymorphic gene with 18 detected alleles. Although the evolutionary conserved *FXC1 *encoded protein has not been directly associated with any human diseases, a loss of function mutation in a related component of the mitochondrial hetero-oligomeric translocase complex, TIMM8A, has been linked with the neurodegenerative disorder, Mohr-Tranebjaerg Syndrome [53]. The extensive protein-protein interactions occurring in the inner mitochondrial membrane translocase complex suggest that variation in FXC1 protein sequence caused by polymorphisms in its poly-Q region may have potential for impacting mitochondrial function [55]. Alternatively, the positioning of the poly-Q tract near the N-terminus of this small protein may have insulated its primary functions from the influence of polymorphic variation.

The number of poly-Q encoding genes in the cow (178) was similar to human (201) especially taking into account that the former number was determined using a draft genome assembly which is missing representation of all genomic sequence (~90% coverage) and does not have completed gene models for all genes [19]. Surprisingly, there was only 61% (109/178) gene overlap between cow and human indicating substantial differences between the species, although the functional enrichments in both groups were similar [11]. The figure of 201 human poly-Q encoding genes (Q ≥ 5) is consistent with an independent figure of 158 human poly-Q genes (Q ≥ 4) in a dataset of 6,477 vertebrate orthologs [37]. However, the subset of 123 bovine CAG poly-Q genes (≥ (CAG)_5_) was substantially larger than that estimated for humans (66-75) and rodents (53-58) [2,11,12]. This observation may partially reflect the impact of the large expansion of CAG repeats in cow genomic DNA compared with other mammalian species (~10 fold increase compared with humans) [19]. As the scale of the repeat expansions in bovine coding sequences is much less than the genome-wide figure, it is likely that coding sequence CAG repeats are subject to different selective pressures compared with non-coding intergenic CAG repeats, as has been previously reported [13,48].

A total of 26 bovine poly-Q encoding genes did not have murine and human orthologs with poly-Q tracts. This group of genes potentially highlights evolutionary adaptations that contribute to the unique biology of ruminants. While some of these encoded proteins contained unique poly-Q tracts, others were identified because there were no orthologs in either the mouse or human genomes or the protein models did not contain sequence corresponding to the orthologous region of the bovine poly-Q tract proteins. The latter case may reflect species specific differences in alternative splicing. More trivial explanations include potentially incomplete gene and protein models or that the Q-tracts in the human and murine orthologs did not reach the threshold size of five residues used to define a Q tract. Notably, these 26 genes were not different from all bovine poly-Q encoding genes in relation to encoded protein size or function.

Comparison of bovine and human poly-Q proteins revealed that the lengths of ortholog Q-tracts were generally similar (Figure 1). This result is different from the finding that rodent Q-tracts were generally shorter than human Q-tracts [2]. As the maximum length of the bovine CAG Q-tract was directly related to allelic diversity (Figure 3) then it may be concluded that the overall extent of polymorphic variation in the poly-Q encoding genes of cattle is likely to be similar to human populations.

Poly-Q encoding genes can potentially generate considerable transcriptional diversity through a variety of mechanisms. The tissue-specific mRNA splicing of *NFYA *alters the length of the poly-Q encoded region in the absence of polymorphic variation. The poly-Q region is part of the activation domain of this transcription factor and modifications to the region have marked effects on function [33]. Thus, the tissue specific splicing may be a means of generating NFYA functional variants which could impact the transcription of the larger repertoire of genes that are regulated by NFYA. Although no poly-Q tract polymorphic variants in NFYA were demonstrated in the current analysis, if they existed, such allelic variants could exert additional tissue specific functional influences through this splicing mechanism. Polymorphic repeat regions in other poly-Q encoding genes traverse splice sites (e.g. *ABCF1*) or are closely juxtapositioned with splice sites (e.g. *MED12*). Moreover, a considerable number of poly-Q encoding genes contained repeats immediately adjacent to or at splice sites. In the case of *MED12 *this was associated with considerable transcriptional variation likely caused by the influence of coding sequence CAG repeats on splicing. There are similarities between CAG repeats and splice donors (consensus sequence (A/C)AG at the 3' end of the exon) and splice acceptors (consensus sequence (C)AG at the 3' end of the intron). This similarity could cause additional mRNA splicing variation in poly-Q encoding genes. Notably the protein MBNL1 (muscleblind-like) is involved in pre-mRNA splicing site choice and it also binds strongly to transcribed CAG repeats [56]. This dual function reinforces the view that splice site choice can be influenced by coding sequence CAG repeats. The presence of poly-Q encoding tracts contained within small alternatively spliced exons for a considerable number of poly-Q encoding genes suggests that this is another mechanism which generates considerable transcriptional and functional diversity. Indeed, the latter is accentuated by the large size of many poly-Q encoding genes, which often encode multiple autonomous functional domains each encoded by discrete exons. Alternative splicing of these exons is frequent and can generate many combinatorial mRNAs for each of these genes, possibly producing a range of protein products with subtle functional variations and a diversity of tissue specific variants.

The bovine poly-Q proteins were strongly enriched for large multi-domain transcriptional regulators (Additional file 4). Moreover, many of these proteins participate in large and common physical interaction networks as well as an extensive network associated with their transcriptional regulation (Figure 4). The central importance of CREBBP in this nuclear network is emphasised by its ability to act as a transcriptional co-activator in conjunction with EP300 to help assemble large regulatory protein complexes at sites of active transcription. Both of these proteins have intrinsic histone acetylase activity, which is involved in modifying chromatin structure in nucleosomes and thereby regulating gene expression [53]. The broad tissue expression patterns of the proteins in these nuclear complexes indicate that they are co-expressed in cells and hence these regulatory networks are likely to be biologically operational, with their components acting cooperatively in large transcriptional regulatory complexes. There could be even greater functional complexity inherent in these complexes caused by polymorphic variation in some of these proteins. Indeed, there is potential for amplification of the phenotypic impact of poly-Q polymorphic variations of multiple components in this regulatory complex - an epistatic effect in genetics terms. Even in the absence of polymorphic variation, alternative splicing influenced by CAG tracts may generate poly-Q protein functional diversity that could impact the activities of large transcriptional regulatory complexes in the nucleus. This process could generate additional functional complexity tailored to the needs of specific tissues, developmental pathways or tissue responses to external stimuli.

## Conclusions

A total of 178 poly-Q encoding genes have been identified in the bovine genome. By using a hierarchical prioritization process we established that at least 16 of 32 top ranked genes were polymorphic in regions encoding poly-Q tracts. There was significant correlation between the extent of allelic diversity and the length of the poly-Q tract. This information, in conjunction with the purity of the repeat region encoding the poly-Q tract and the lack of conservation of repeat length, provide indicators for the propensity for polymorphic variation in the full repertoire of poly-Q encoding genes. Unlike some human poly-Q encoding genes there was no evidence for repeat instability, which may be attributable to the lack of large repeat expansions in the normal cattle population. This may be an inherent biological characteristic of cattle or a reflection of strong selective pressures in breeding programs that exclude these variants. Polymorphic variations in bovine poly-Q proteins have strong potential to generate epistatic biological effects due to the involvement of these proteins in transcriptional regulatory complexes and protein-gene regulatory interactions. Thus, the bovine poly-Q encoding genes may be contributing to phenotypic variation in cattle populations both directly and indirectly through epistatic interactions. CAG repeat tracts in poly-Q encoding genes can also influence splice site choice and thereby increase the functional diversity of transcripts from these genes.

## Methods

### Animal resources

#### Large cattle diversity panel

*Bos taurus taurus *animals (150) were represented by seven dairy breeds (Australian Red Breed (n = 16), Ayshire (n = 3), Brown Swiss (n = 3), Guernsey (n = 7), Holstein Friesian (n = 24), Illawarra Shorthorn (n = 12) and Jersey (n = 15)) and five beef breeds (Angus (n = 8), Belgium Blue (n = 3), Charolais (n = 4), Poll Hereford (n = 11) and Shorthorn (n = 9)). The *Bos taurus indicus *animals (35) included four breeds (Africander (n = 7), Boran (n = 8), Brahman (n = 12) and Tuli (n = 8)) (Additional file 2). The Australian Dairy Herd Improvement Scheme (ADHIS) database was used to ensure that the most unrelated dairy animals available to us were included in the panel; while other animals were sourced from diverse properties throughout Queensland, Australia.

#### Small cattle diversity panel

Eight animals were selected from the large diversity panel for initial screening for polymorphic variation. These animals included one of each from the Shorthorn, Poll Hereford, Boran, Tuli, Africander, Brahman, Holstein Friesian and Charolais breeds.

### Identification of bovine genes encoding poly-Q tracts

Tracts of five or more glutamines (Q-tracts) encoded by bovine genes were identified using the following procedure. Cattle protein sequences in NCBI's RefSeq database, which were derived from annotation of the Btau 3.1 genome sequence assembly [19,20], were down-loaded (ftp://ftp.ncbi.nih.gov/genomes/Bos_taurus/protein/protein.fa.gz). Within this set 178 proteins containing at least one Q-tract were identified using a simple script. This analysis was performed without regard to whether glutamine was encoded by CAG or CAA codons. The genes were also manually re-examined using the Btau 4.0 assembly using both RefSeq and Ensembl gene models to confirm Q-tracts and the nature of their coding sequences. Reciprocal mapping of human and bovine orthologs was also performed (Additional file 1). This highlighted human orthologs that map to more than one GLEAN5 model, which may represent local assembly issues in the bovine genome assembly and/or incomplete gene models. Only unique bovine GLEAN5 models were used.

To identify evidence of poly-Q tract length variation in sequence databases due to possible allelic contributions, the 178 protein sequences were used as queries in tBlastn searches run with NCBI's *Bos taurus *genomic Blast service [18]. The Expect cut-off value was set to 0.00001 and default values were used for other parameters. Two databases were queried: *Bos taurus *HTGS (High Throughput Genome Sequence) and *Bos taurus *expressed sequence tags (ESTs). Results with a maximum of 50 alignments were formatted as 'query anchored' HTML and scrutinised for alignments in which there was variation in the length of a poly-Q tract. After excluding non-allelic variation (splicing in the case of the EST database searches, and gene paralogy in the case of the HTGS database searches), 19 candidate proteins were identified (NFYA, FXC1, FAM48A, CACNA1A, FOXP1, FAM155A, ABCF1, MLL3, NUFIP2, CREBBP, FOXP2, GLG1, MED12, LRRC4, FBXO11, ST6GALNAC5, THAP11, NCOR1, MED15). Throughout the text gene symbols were italicised except when used in the context of their encoded proteins.

### Prioritization of bovine genes for experimental analysis of polymorphic variation in regions containing poly-Q tracts

A number of characteristics have been associated with propensity for polymorphic variation in genes containing trinucleotide repeats encoding poly-Q tracts [11]. The bovine genes encoding poly-Q tracts were hierarchically prioritised according to the following criteria: (i) length of pure CAG repeat encoding a poly-Q tract (≥(CAG)_5_); (ii) lack of conservation of the poly-Q tract lengths in mammalian orthologs (manual inspection using HomoloGene Release 64; [18]); (iii) evidence for polymorphic variation in other mammalian species [1,8,11,17]; (iv) demonstration that polymorphic variations in the human or mouse orthologs caused diseases [1,8,11,17], and; (v) apparent disparities between bovine cDNA sequences and the bovine reference genome sequence in genes encoding poly-Q repeats. Genes in each category were assigned a value of 1 or 0 and ranked according to their cumulative category score. Genes with identical scores were then ranked according to CAG repeat length. The top 32 ranked genes selected according to criteria (i) to (iv) also included 16 of the 19 genes identified by criterion (v). The 32 genes were screened for the presence of polymorphic variation in their CAG repeat regions using the small cattle diversity panel consisting of eight animals. Some genes were assessed for multiple repeat regions. Genes that showed evidence of polymorphic variation in the small diversity panel were then assessed in the larger cattle diversity panel. Also included in both analyses were the genes *AR*, *ODAM *and *NFYA*, representing genes that were lowly ranked in the prioritisation process and therefore predicted to be monomorphic.

### DNA purification

Genomic DNA was extracted from white blood cells using 200 μl of whole blood and the QiAmp DNA Mini kit (Qiagen). DNA quality was assessed as an A_260_/A_280 _ratio greater than 1.8. The DNA was quantified by spectrophotometry and diluted to a working concentration of 10-50 ng/μl with 10 mM Tris-HCl, pH 7.5, 1 mM EDTA.

### Genotyping

#### PCR screening

Preliminary PCR analysis of 32 prioritised genes and the 3 low ranking genes containing poly-Q tracts was undertaken using DNA from the small diversity panel. Each gene was subjected to PCR to generate an amplicon traversing the region(s) containing the repeats encoding the poly-Q tract. Some genes contained more than one region and these were all analysed. Primers were designed from bovine genome sequence information (Btau4.0; [19] (Additional file 6). PCR reactions (10 ml) contained 10-50 ng genomic DNA, 0.5 μM of each primer, 200 μM dNTP in 1 × Reaction Buffer (1 × Q solution) and Hot-Star Taq (Qiagen). The PCR conditions were 94 °C for 15 min, followed by a touch-down protocol: (11 initial cycles of 94 °C for 30 s, 65-57°C for 30 s with a reduction of 0.7°C/cycle, and 72°C for 45 sec; 32 cycles of 94°C for 30 s, 57°C for 30 s and 72°C for 45 s; and a final extension step at 72°C for 15 min). The amplified product was visualised on 3% agarose gels stained with ethidium bromide. Controls containing no sample were routinely performed. The amplified DNA products were sequenced to confirm gene identity and to provide size references for the repeat regions identified by multiplex genotyping.

#### Multiplex genotyping assays

Nineteen of the initial 32 prioritised genes showed evidence of polymorphic variation in the initial PCR screen using the small diversity panel. Multiplex genotyping assays were then developed for these genes so they could be analysed on an ABI 3130 × l Genetic Analyser (Applied Biosystems, Australia). Forward primers were labelled with the fluorophores specified in Additional file 6 and three sets of markers were developed for the efficient genotyping of all 19 genes. The first set included *RUNX2*, PCR amplified as a singleplex and (*ST6*, *FAM48A*, *MLLQ3 *and *FXC1*) amplified as a multiplex; the second set included two PCR multiplexes (*CREBBP*, *NCOR1*, *ABCF1 *and *MED12-Q1*) and (*THAP11*, *HTT *and *MED15-Q1*)); while the third set comprised (*C10ORF26*, *EXDL2*, *TBP*) and (*FAM55A*, *MEF2A*, *CACANA1 *and *LRCH4*) amplified as two separate multiplexes. The suffix '-Q1' attached to a gene symbol refers to the first of more than one poly-Q encoding region within a gene. Each PCR product (0.5 μl of a 1:100 dilution) from each marker set was co-loaded onto the ABI 3130 × l Genetic Analyser for capillary electrophoresis according to the manufacturer's instructions with LIZ 500 (Applied Biosystems) as the size standard. Sizing of PCR products was accomplished using GeneMapper software (v4.0, Applied Biosystems). PCR reactions (10 ml) contained 10-50 ng genomic DNA, 0.1 μM of FAM-labelled primer pair, 0.25 μM of VIC-, NED- and PET-labelled primer pairs, 1× Q solution and 1 × reaction buffer (Qiagen Multiplex PCR Kit). Multiplex PCR conditions were 94 °C for 15 min; followed by a touch-down protocol: 11 cycles of 94 °C for 30 s, 65-57°C for 1 min 30 s with a reduction of 0.7°C/cycle, and 72°C for 45 s; 32 cycles of 94°C for 30 s, 57°C for 1 min 30 s, and 72°C for 45 s, and; a final extension step at 72°C for 15 min. PCR conditions were similar for singleplex reactions except that Hot-Star Taq (Qiagen) and 200 μM dNTP were used in the reaction and the annealing time in the touch-down protocol was reduced to 30 s.

### cDNA synthesis and reverse transcription PCR (RT-PCR)

Total RNA was extracted from a range of bovine tissues using Trizol reagent (Invitrogen) followed by DNase I (Ambion) treatment [57]. RNA was quantified, its quality verified and cDNA synthesized using an anchored oligo-T_18 _primer combined with random hexamers [57]. To validate the absence of genomic DNA, control experiments were performed without reverse transcriptase. cDNA derived from each bovine tissue sample was subjected to PCR for specific amplification of *NFYA*. The FAM-labelled sense and antisense primers were designed to traverse the region encoding the poly-Q sequence (sense primer, 5'-CAAACAGCAACAGTTCAGCAG-3'; antisense primer, 5'-CAGGGTCTGGACTTGCTGG-3'). The conditions used for PCR were as described above. The amplified product was sequenced to confirm its identity. All samples contained the same quantity of first-strand cDNA (0.9 ng). Controls included no template or sample that was not subjected to cDNA synthesis using reverse transciptase. In addition, PCR was undertaken using genomic DNA but with *NFYA *primers spanning the exon 2 - intron 2 and intron 2 - exon 3 boundaries (sense primer, 5'-CAAACAGCAACAGTTCAGCAG-3'; antisense primer, 5'-AGAAGATCCGATGCCTCTCA-3': and sense primer, 5'-GAAGACCCTTCCTCTCCCAC-3'; antisense primer, 5'-CCTCAGTCTGCAACTGGACA-3', respectively).

### Functional annotations

To identify higher level functional themes associated with the bovine poly-Q encoding genes, they were analysed by using the Database for Annotation, Visualization and Integrated Discovery (DAVID) with a background containing all genes represented in the bovine genome sequence including Chromosome Unknown [26,27]. DAVID provides statistical methods for identification of enriched biological terms within data sets. Statistically over-represented Gene Ontology (GO) terms, keywords and pathways were identified by selecting those with a Benjamini-adjusted p-value < 0.05. Similar results were obtained by using the full complement of human genes as background. *Functional Annotation Clustering *was then performed using the DAVID system. This higher level analysis displayed similar functional annotations together based on overlaps of genes associated with each function term and therefore gives a clearer overview of gene function information associated with large datasets. In this instance, enrichment scores (- log (geometric mean of the P-values for terms in the cluster)) greater than 1.3 were considered significant. The conservative *High Stringency *option was used in conjunction with default settings for other parameters. Analysis of the subgroup of bovine genes containing poly-Q tracts which were not present in their human and murine orthologs used the same analysis procedure except that the background was all bovine genes containing poly-Q tracts.

### Interaction networks

The InnateDB database was used to identify networks of physically interacting poly-Q proteins using their human RefSeq orthologs as input [28,29]. The database contains comprehensive records of protein-protein and protein-gene interactions that have been experimentally defined, as well as links to relevant PubMed records [18]. The interactions were restricted to only those within the group of submitted proteins and were visualised using Cerebral, which is a Java plugin for the Cytoscape biomolecular interaction viewer [58]. Proteins that regulated the expression of genes in the list were also included and their links were colored red.

## Authors' contributions

RT conceived and designed the experiments, analysed the results and wrote the manuscript. VW performed the gene prioritization and genotyping. MH, RT and YS-L identified and annotated the bovine poly Q encoding genes. WB provided DNA samples from a population of cattle while MK and HR supplied trio samples. All authors read and approved the final manuscript.

## Supplementary Material

Additional file 1 **Bovine poly-Q encoding genes**. This file contains details of the bovine poly-Q encoding genes including gene symbols, gene and protein accessions, GLEAN5 accessions [19], the number, sizes and purities of poly-Q tracts encoded in each gene and corresponding annotation information for human orthologs. The reciprocal mapping highlighted human orthologs that map to more than one GLEAN5 model which may represent local assembly issues in the bovine genome assembly, incomplete gene models or inability to discriminate between paralogs. Also included are multiple accessions for some bovine genes which are used to discriminate between splice variants. Bovine genes containing poly-Q tracts that were not present in their human or murine orthologs are bolded. The purity of each tract was defined by a combination of G (for CAG) or A (for CAA) symbols followed by numbers. Genes that were assessed for polymorphic variation in the large cattle diversity panel are also listed with the number of identified alleles. Click here for file

Additional file 2 **Large cattle diversity panel**. The breed composition of the large cattle diversity panel is shown. Also included is a description of whether the *Bos taurus taurus *breed was primarily used for beef or dairy production. Click here for file

Additional file 3 **Relative distributions of allele frequencies**. The allele frequency distribution for each polymorphic poly-Q encoding gene is presented as a histogram. Each allele was defined by amplicon size. In a few instances differences in amplicon sizes did not seemingly differ by a multiple of three nucleotides, as expected for trinucleotide repeat polymorphisms. This observation may reflect non-trinucleotide repeat indels or could be caused by the calibration process used to measure amplicon size. Where alleles were sequenced they were consistent with the latter explanation. Click here for file

Additional file 4 **Functional clustering of bovine poly-Q encoding genes**. Summary table listing enriched Functional Annotation Clusters and functional terms significantly associated with the bovine poly-Q encoding genes. The analysis was performed using DAVID analysis tools [26]. The conservative *high stringency *option was elected. P-values were corrected for multiple testing using the Benjamini correction. The enrichment score for a Functional Annotation Cluster is the -log (geometric mean of the term P-values within the cluster). An enrichment score ≥1.3 was considered significant. Click here for file

Additional file 5 **Genomic organisation of two adjacent exons in *MED12 *that encode poly-Q tracts**. UCSC bovine genome browser [22] representations of the 3' and 5' ends of two adjacent exons in bovine *MED12*. The exons are separated by an ~ 820 bp intron. Transcription is from right to left. Panel (a) is the 5' exon and panel (b) is the 3' exon of the gene. Exon coordinates are listed in each panel. Both exons encode large poly-Q tracts. Also included is protein sequence information for cow, dog, human and mouse as well as informative bovine ESTs. A full description of each track can be found at the UCSC genome browser. Click here for file

Additional file 6 Primer sequences used for genotyping Click here for file
