# Supplementary material for: Bovine proteins containing poly-glutamine repeats are often polymorphic and enriched for components of transcriptional regulatory complexes
Source: BMC Genomics. 2010 Nov 23;11:654. doi: 10.1186/1471-2164-11-654 (PMC3014979; doi:10.1186/1471-2164-11-654)
Supplement: Additional file 2 — Large cattle diversity panel. The breed composition of the large cattle diversity panel is shown. Also included is a description of whether the Bos taurus taurus breed was primarily used for beef or dairy production. [file 1471-2164-11-654-S2.DOC]

**Additional file 2**. Breed composition of the large cattle diversity panel

| **Breed** | **Animals assayed**  **(number)^1^** | ***Bos taurus taurus* or *Bos taurus indicus breed*** | ***Bos taurus taurus* Beef or Dairy** |
| --- | --- | --- | --- |
| Africander | 7 | *Bos taurus indicus* |  |
| Boran | 8 | *Bos taurus indicus* |  |
| Brahman | 12 | *Bos taurus indicus* |  |
| Tuli | 8 | *Bos taurus indicus* |  |
| Australian Red | 16 | *Bos taurus taurus* | Dairy |
| Ayshire X | 3 | *Bos taurus taurus* | Dairy |
| Brown Swiss | 3 | *Bos taurus taurus* | Dairy |
| Guernsey | 7 | *Bos taurus taurus* | Dairy |
| Holstein Friesian | 24 | *Bos taurus taurus* | Dairy |
| Illawarra Shorthorn | 12 | *Bos taurus taurus* | Dairy |
| Jersey | 15 | *Bos taurus taurus* | Dairy |
| Angus | 8 | *Bos taurus taurus* | Beef |
| Belgium Blue | 3 | *Bos taurus taurus* | Beef |
| Charolais | 4 | *Bos taurus taurus* | Beef |
| Poll Hereford | 11 | *Bos taurus taurus* | Beef |
| Shorthorn | 9 | *Bos taurus taurus* | Beef |

^1^A total of 150 cattle were examined.
