# Supplementary figures and images for: Bovine proteins containing poly-glutamine repeats are often polymorphic and enriched for components of transcriptional regulatory complexes
Source: BMC Genomics. 2010 Nov 23;11:654. doi: 10.1186/1471-2164-11-654 (PMC3014979; doi:10.1186/1471-2164-11-654)

## Slide 1
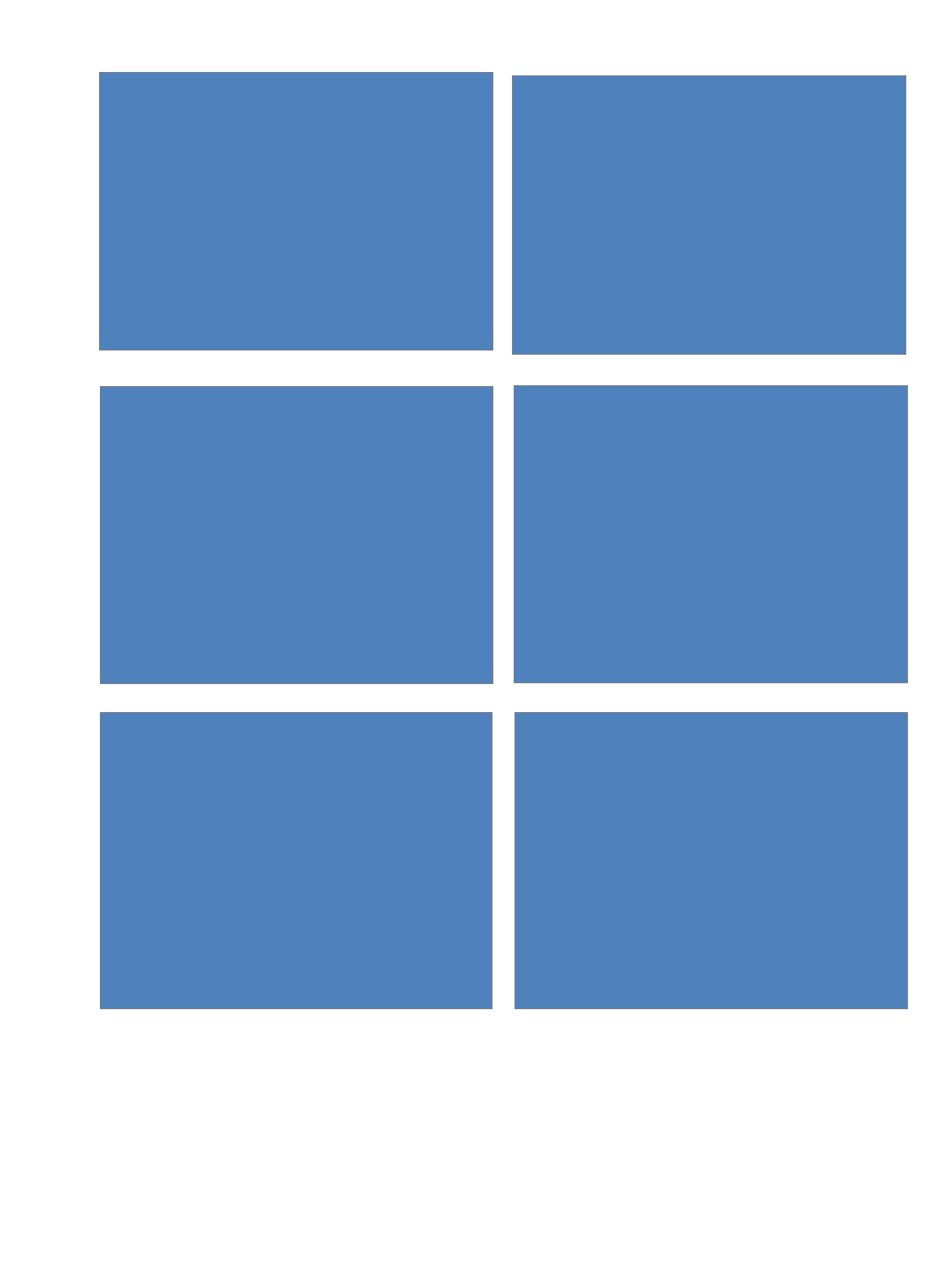

## Slide 2
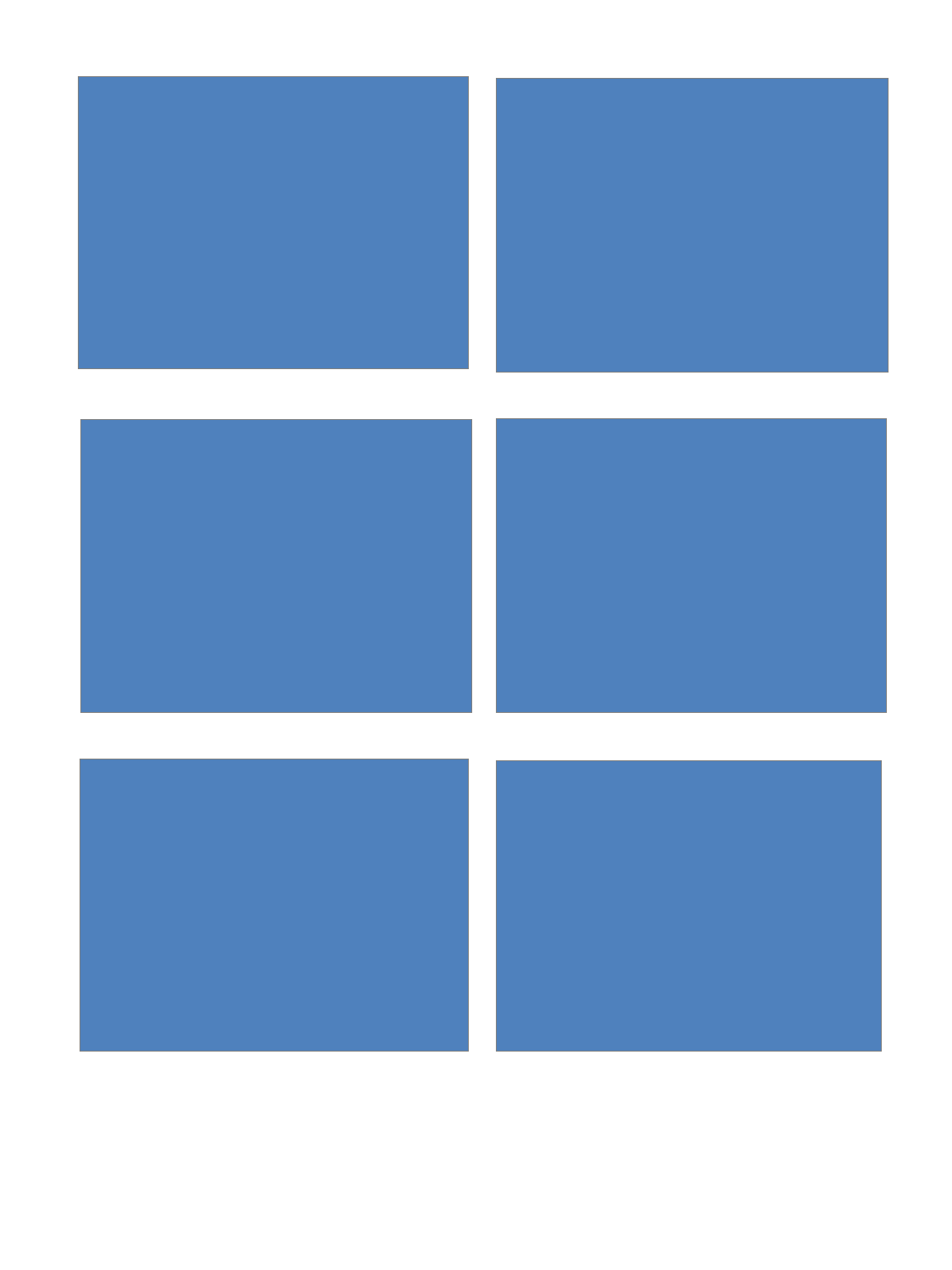

## Slide 3
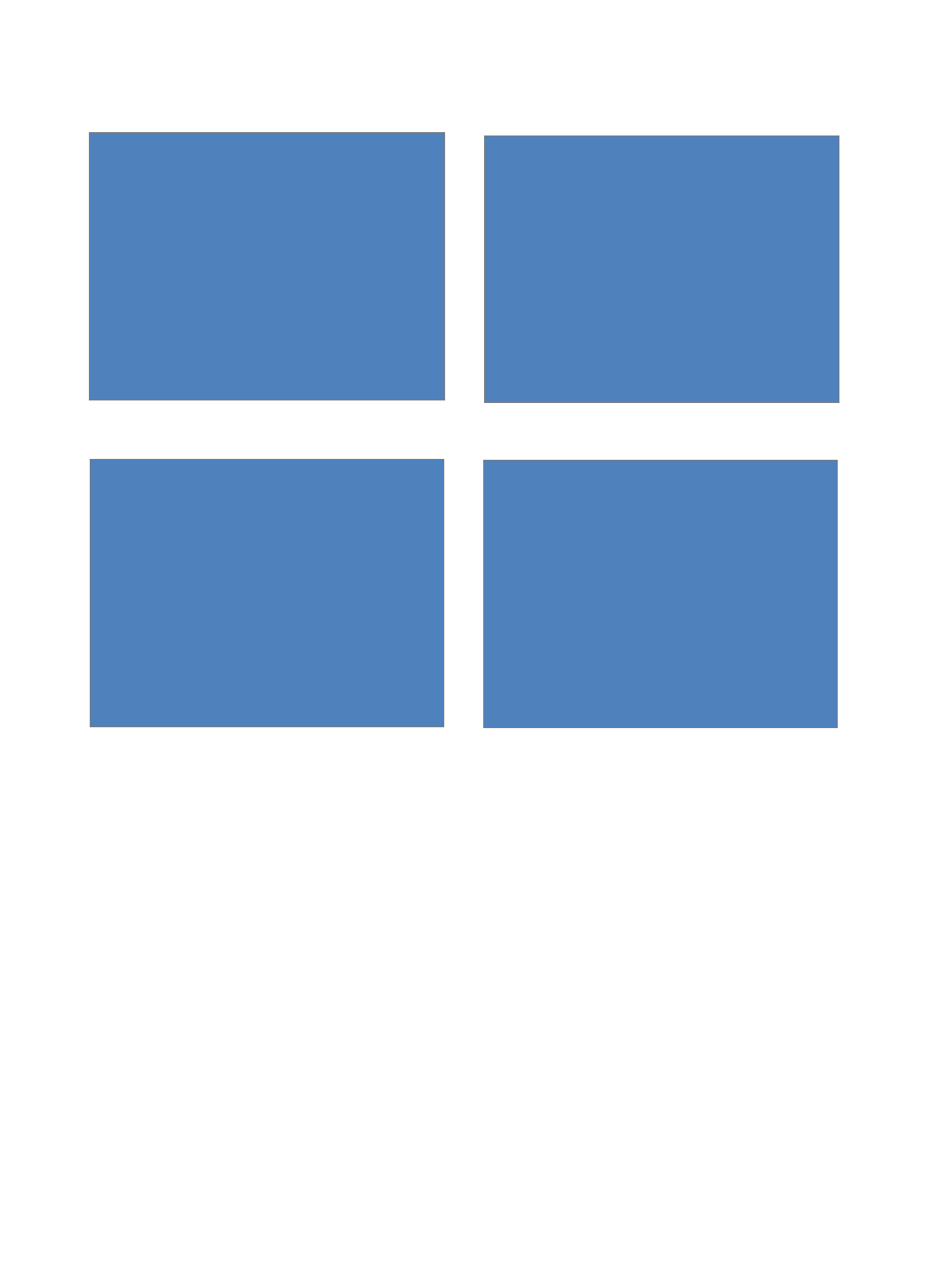

Supplement: Additional file 3 — Relative distributions of allele frequencies. The allele frequency distribution for each polymorphic poly-Q encoding gene is presented as a histogram. Each allele was defined by amplicon size. In a few instances differences in amplicon sizes did not seemingly differ by a multiple of three nucleotides, as expected for trinucleotide repeat polymorphisms. This observation may reflect non-trinucleotide repeat indels or could be caused by the calibration process used to measure amplicon size. Where alleles were sequenced they were consistent with the latter explanation. [file 1471-2164-11-654-S3.PPT]

## Slide 1
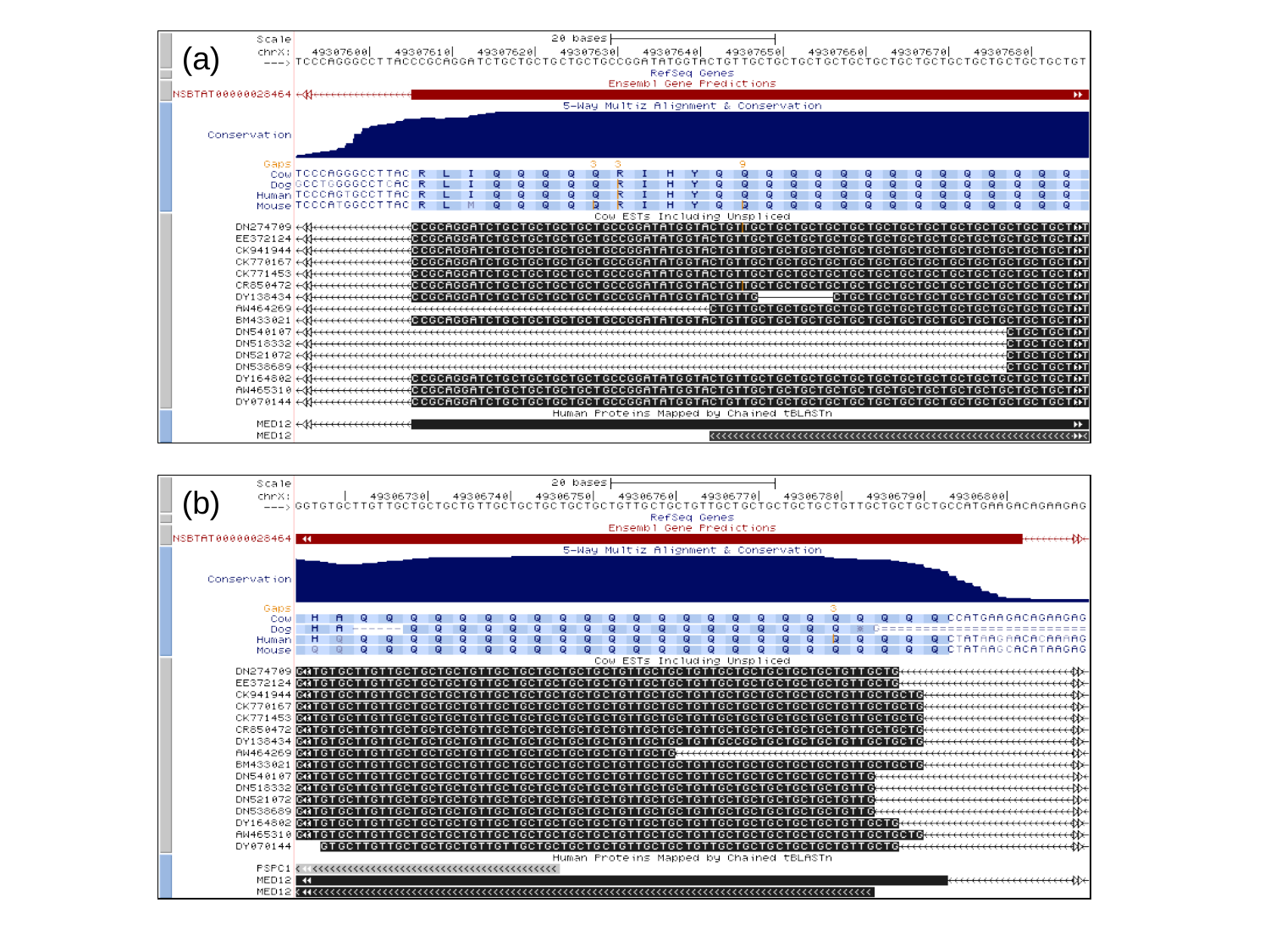

(a)
(b)

Supplement: Additional file 5 — Genomic organisation of two adjacent exons in MED12 that encode poly-Q tracts. UCSC bovine genome browser [22] representations of the 3' and 5' ends of two adjacent exons in bovine MED12. The exons are separated by an ~ 820 bp intron. Transcription is from right to left. Panel (a) is the 5' exon and panel (b) is the 3' exon of the gene. Exon coordinates are listed in each panel. Both exons encode large poly-Q tracts. Also included is protein sequence information for cow, dog, human and mouse as well as informative bovine ESTs. A full description of each track can be found at the UCSC genome browser. [file 1471-2164-11-654-S5.PPT]
