# Supplementary material for: Bovine proteins containing poly-glutamine repeats are often polymorphic and enriched for components of transcriptional regulatory complexes
Source: BMC Genomics. 2010 Nov 23;11:654. doi: 10.1186/1471-2164-11-654 (PMC3014979; doi:10.1186/1471-2164-11-654)
Supplement: Additional file 6 — Primer sequences used for genotyping [file 1471-2164-11-654-S6.DOC]

**Additional file 6. Primer sequences used for genotyping**

| **Gene Symbol^1^ (poly Q region)** | **Forward primer (5'-3')** | **Reverse primer (5'-3')** | **Fluorophor (ABI 3130xl Genetic Analyser)** | **Expected fragment size from reference genome (BTAU 4.0)** |
| --- | --- | --- | --- | --- |
| ***ABCF1*** | GCTGGGGAAGAAGAGAAAGTG | GAAAGCCAAACAGGGTGAGA | FAM | 225 |
| *AR* (Q1) | AGTGATCCAGAACCCACTCC | TGAAGGCTGCTGTTCCTCTT |  | 196 |
| *AR* (Q2) | CGGCACCATGCAACTTCT | GGACACCGACACTGCCTTAC |  | 181 |
| *ATN1* | AGGTCCCTTCCCTCCTTCAG | AGACATGGCATAGGGGTGTG |  | 205 |
| *ATXN2* | CCTCACCATGTCGCTGAA | TGAGGAGGAGGAGGAAGAAG |  | 178 |
| *ATXN3* | TGATTGCTCTTGCCTTCTTTG | AGCTTGTGCTGGACTTTTCAC |  | 223 |
| *BACCH1* | TGATGTCATGGACCAGGCT | GGTGGGTACCTGGAATTGG |  | 220 |
| ***C10orf26*** | ATGAGGAAGTGGTGAACCGA | GTCGCGTGCTACTTCTGCT | NED | 198 |
| ***CACNA1A*** | GTGTCCTATTCCCCCGTGAT | CTGCTTCCGTGGCCTCC | VIC | 170 |
| ***CREBBP*** | TACCGAGAGATGCTGAGGAG | CTGCTGGAACTGGCTGTG | FAM | 126 |
| ***EXDL2*** | CCTTGACAGTGACCACCCTT | ATCCTCTCTTCCCATGAGGC | FAM | 223 |
| ***FAM155A*** | GCTCTCTGATCACTTGTGGTTCT | AGGCGGAGAGGAGTCTGTG | VIC | 218 |
| ***FAM48A*** | GGCTCGCTCATTTTTAACACTC | GGACTAGAAGTTGTGGGTTGCT | FAM | 107 |
| *FBX011* | GGAGACCCAGGCGAGTGT | AAAGAGCGCGGCCTTACC |  | 218 |
| *FOXP1* (Q1) | GATGCAAGAATCTGGGACTGA | GTGTGCGATGGAACGGTAG |  | 240 |
| *FOXP1* (Q2) | CTCATCCTCATGTGTCCTTCCT | TGTCATTCCTCTTGGGAGACTT |  | 181 |
| *FOXP2* | CAGCAAGAGCAGTTACATCTTCA | CCTACCTCTTTTGCTTGCTTTC |  | 170 |
| ***FXC1*** | CCAAGAACCTGAAAAGGAAATG | AGACGACTTAGCTCCCACTCAC | VIC | 135 |
| *GLG1* | GCTGCCTCAGTCATCTCAGT | TTGCTCCAGGTGTGCTTG |  | 228 |
| ***HTT*** | CGACCCTGGAAAAGCTGATG | GGCTGAGGGGGCTGAGG | PET | 130 |
| ***LRCH4*** | ACACCTTGCAGCTGTGGC | CACCCCCAAGAGACAGAGAA | FAM | 162 |
| *MAML2* | TTTAACTCGGATCAAGCCAAC | TCTTTTGCTGCAGTGGTAATG |  | 289 |
| ***MED12* (Q1)** | AGCAAACACCCATGATAGGC | ATGAAATCCCAGGGCCTTAC | VIC | 223 |
| *MED12* (Q2) | CTGTGCTTCCTCTGCTCTCC | AATGTTCCCCATCCTTCTCC |  | 356 |
| ***MED15* (Q1)** | GTCTGTCTCCAGCCCAGC | CCTGGACGTGCTCAGAGG | PET | 208 |
| *MED15* (Q2) | GCTGATGGGGTTGCTTATTC | AAGGCTGTGGCTGTGACTG |  | 236 |
| ***MEF2A*** | GCTGGAGGGCAGTTATCTCA | CACAACGGGAGAGTGGAAGT | FAM | 282 |
| *MLL3* (Q1) | GAAGCTGGAGCAGGTGAAGA | ATGACTGGGCAGGAGACAGA |  | 176 |
| ***MLL3* (Q3)** | CATGCCGAGTTGATTGAAGA | GTTGCCACCCAGGTAAGC | PET | 238 |
| ***NCOR1*** | AATTTGCGGTCCCTGATTAC | CCGGGTGAAACTCTGAAAGTAG | VIC | 132 |
| ***NFYA*** (cDNA^2^) | CAAACAGCAACAGTTCAGCAG | CAGGGTCTGGACTTGCTGG | FAM | 140 |
| *NSUN2* | GAGGCGCATGTGCTGAC | GTGAGAGAAACTTGGCACCC |  | 274 |
| *NUFIP2* | CGCTTTCAATGGAGGAGAAG | TCATGTTTCAGCAGCTTTGG |  | 232 |
| ***ODAM*** | TGCTTCCTTTCTGTCTCTTGTG | ACCAGTCCAGCAAACCACTC | FAM | 150 |
| *PHC1* | AGCTTCTGCACACAGCCAC | AGGGACAGCGGTGAAGACT |  | 196 |
| ***RUNX2*** | GGCAAGATGAGCGACGTGAG | CAGCACGGAGCACAGGAAGT | FAM | 261 |
| *SLFNL1* | GTCTCAGCCCTCATCAGCTC | GCACTAGGCCCTCACAAGAC |  | 191 |
| ***ST6*** | CATGGTCTGGCAGTGTGTTT | CCGAGGTATCCGTCCAGT | FAM | 212 |
| ***TBP*** | CCTTACGGCACAGGACTGAC | GGGAGGGATACAAGGGAGTG | VIC | 268 |
| ***THAPP11*** | GTAAACGAGCGCAAAGTAGCA | CTGAAGGGTGAGGAGCACAG | NED | 198 |

^1^Fluorescent genotyping assays developed for some genes are shown in bold. Some genes had more than one poly Q encoded region. Each region is designated in brackets.

^2^Fluorescent assay developed for cDNA (see Methods)
